# Functional Capacity and Inflammatory Mediators in Elderly Residents of Counties with Different Human Development Index

**DOI:** 10.1155/2020/9250929

**Published:** 2020-01-21

**Authors:** Lygia P. Lustosa, Daniele R. Xavier, Giane A. Ribeiro-Samora, Daniele S. Pereira, Adriana N. Parentoni, Ronaldo L. Thomasini, Leani S. M. Pereira

**Affiliations:** ^1^Post-Graduate Program in Rehabilitation Science, Physical Therapy Department, Universidade Federal de Minas Gerais, Belo Horizonte, Minas Gerais, Brazil; ^2^Universidade Federal de Alfenas, Alfenas, Minas Gerais, Brazil; ^3^Medicine Faculty, Universidade Federal dos Vales do Jequitinhonha e Mucuri, Diamantina, Minas Gerais, Brazil

## Abstract

A number of studies have indicated that certain factors, including socioeconomic status and education, are associated with the functional health status of the elderly. Another relevant factor in aging is chronic subliminal inflammation, with increased levels of circulating inflammatory cytokines such as interleukin-6 (IL-6), tumor necrosis factor *α* (TNF-*α*), and soluble tumor necrosis factor receptor 1 (sTNFR-1), commonly seen in the elderly. High levels of these inflammatory mediators could impair the functional capacity. In this respect, the aim of this cross-sectional study was to compare plasma levels of inflammatory mediators and functional capacity of older women living in three Brazilian counties with different Human Development Index. We evaluated 154 women aged ≥65 years, regardless of race and/or social status. IL-6 and sTNFR-1 plasma levels were measured by ELISA and the functional capacity by the Short Physical Performance Battery (SPPB) test. Comparison among groups was performed using one-way ANOVA with Bonferroni post hoc correction, Kruskal–Wallis, and Mann–Whitney *U* tests. Women that lived in one of the counties with high HDI had lower functional capacity (*p* < 0.001). The population from the county with the highest HDI had lower plasma levels of sTNFR-1 (*p* < 0.05). There was no significant difference in plasma levels of IL-6 (*p* > 0.05). Besides this, women from the counties with lower HDI had a higher number of self-reported diseases and higher income (*p* < 0.05). Women that lived in the county with the highest HDI had a higher average education level (*p* < 0.05). The results showed differences in functional capacity and plasma levels of sTNFR-1 between the counties. In addition, the level of education, family income, and number of self-reported diseases show regional diversities in the aging process, suggesting these factors having an influence on inflammatory mediators and functional capacity.

## 1. Introduction

Increased life expectancy is a fact in our society. However, this can only be considered an achievement when it enhances people's quality of life [1]. Among the aspects that are related to a better quality of life in old age, we highlight functional capacity, which can be considered an important marker of health in the elderly [2–4]. In addition to worldwide ethnic diversity, socioeconomic disparities and inequalities in access to education and health care among regions can be observed even within the same country. These differences may have an impact on functional capacity [5, 6].

Indeed, considering regional differences, evidence has shown that a higher level of education in the elderly leads to lower risk of functional impairment. High level of education seems to be a determinant of favorable working conditions, higher income, and better access to health care, all of which positively impact functional capacity [7]. Theoretically, Human Development Index (HDI), which is a measure of health, education, and income may be related to the functional capacity of the elderly.

Another relevant etiologic factor in aging is the inflammatory phenomenon characterized by chronic subliminal inflammatory state with a two- to four-fold increase in plasma levels of cytokines such as interleukin-6 (IL-6), tumor necrosis factor *α* (TNF-*α*), and soluble tumor necrosis factor receptor 1 (sTNFR-1) in older adults [8]. From this, it is assumed that chronically high levels of proinflammatory cytokines cause catabolism in the muscle which contributes to the onset of sarcopenia and subsequent impairment of functional capacity [9, 10]. Thus, it can be expected that differences in levels of inflammatory mediators may exist along with sociodemographic and functional capacity differences. In the same way, observed differences in functional capacity may also determine differences in inflammatory mediators, which can result in a vicious cycle.

In developing countries, the literature clearly points out that the elderly population is composed predominantly of women, often widows, with a low level of education and a low income [1, 11]. These factors may have an impact on functional capacity and on levels of inflammatory mediators. Given the higher life expectancy of women associated with the regional heterogeneities of the aging process, socioeconomic level, educational and clinical differences, it is suggested that comparing functional capacity of older women from different counties with different HDI can contribute to further knowledge of the functional capacity and the inflammatory stage of these older women [2, 5, 9, 10]. Thus, it is believed that studying possible differences in the functional capacity and plasma indices of inflammatory mediators in women living in cities with specific characteristics may increase knowledge about these interrelationships. Therefore, in this study, we hypothesized that older women living in cities with very high HDI were in better functional capacity and had lower levels of inflammatory mediators.

The objective of the present study was to compare the plasma levels of inflammatory mediators (IL-6 and sTNFR-1) and functional capacity among community-dwellings of older women living in three counties with different HDI, classified as very high and high.

## 2. Methods

### 2.1. Study Design

This was a cross-sectional observational study, as part of a multicenter project to evaluate older women in counties with different HDI. The HDI is a summary, a comparative measure used to rank countries and counties according to their degree of human development [6]. Long-term progress in three basic dimensions of human development, income, education, and health, are considered to calculate the index. More specifically, it uses education (literacy and enrollment), longevity (life expectancy at birth), and income indicators [6]. For this study, three counties were considered: one with a very high HDI (0.810) and two others with a high HDI (0.761 and 0.716) but with some sociodemographic differences [6]. The counties chosen are from the same state (Minas Gerais); the one with a very high HDI is the capital and the other two are important touristic and industrial cities in regions of this state. The choice of counties was not probabilistic but based on the fact that they are university sites and important research centers. All participants were informed of the study procedures and signed the consent form which had been approved by the Research Ethics Committee. Data collection took place from October 2015 to January 2017.

### 2.2. Sample

Women aged ≥65 years, living in three Brazilian counties with different HDI—very high HDI (0.810) and high HDI (0.761, 0.716) [6]—and without ethnic and/or social class distinctions participated in the study. Participants who presented cognitive impairment, as assessed by the Mini Mental State Examination, which takes into account participants' level of education, were excluded [12]. Clinical evaluation and medical record information were investigated, and patients with the following conditions were also excluded from the study: acute phase of inflammatory disease which would be a contraindication to performing the tests; exacerbated cardiovascular and metabolic diseases; active cancer in the previous five years; fracture or hospitalization in the last year; use of anti-inflammatory or immune-mediated drugs; inability to walk independently; neurological changes and sequels.

### 2.3. Characteristics of the Study Population

In order to characterize the sample, a standardized questionnaire was used to collect the sociodemographic data and information on clinical conditions, such as age, marital status, ethnicity, level of education, personal income, number of comorbidities and medications, presence and intensity of pain, and life self-satisfaction.

### 2.4. Procedures

Participants were recruited by active search in reference centers for the elderly. Eligible participants were evaluated by trained researchers. Initially, peripheral blood collection was performed by a nursing assistant, followed by an interview with questions on clinical and sociodemographic characteristics and performance of the Short Physical Performance Battery (SPPB) test [13, 14].

### 2.5. Blood Collection and Quantification of Plasma Levels of IL-6 and sTNFR-1

Peripheral blood samples were collected between 8 and 10 am using a commercial vacuum system with ethylene diamine tetracetic anticoagulant (EDTA) (Vacutainer®, BD Biosciences, Franklin Lakes, NJ, USA). Plasma separation was done immediately after centrifugation and then frozen at −80°C. Plasma concentrations of IL-6 and sTNFR-1 were measured by enzyme-linked immunosorbent assay (ELISA) using high-sensitivity kits (Quantikine®HS, R & D Systems, Minneapolis, USA) and Duo Set® (Human sTNF RI/TNFRSF1A), respectively. All procedures were carried out following the manufacturer's instructions.

### 2.6. Functional Capacity

The functional capacity was evaluated by the SPPB, composed of three stages: static balance, gait speed, and estimated muscular strength of lower limbs. The test started with the evaluation of the static balance (feet side by side, posture semi tandem, and tandem). Subsequently, the gait speed test, in the course of four meters, was carried out without considering acceleration and deceleration. Finally, the sit-up chair was tested for five consecutive times, with arms crossed in front of the trunk. All the tests were timed, and the final score was used for analysis. The intraclass correlation analysis of SPPB demonstrated a high level of interobserver reliability (CCI = 0.996) [13, 14].

### 2.7. Statistical Analysis

The sample size calculation was based on a pilot study with 10 older women from each county, considering the functional capacity for a nondirectional analysis, 95% confidence interval, estimated maximum value for alpha error of 5%, maximum value estimated for the beta error of 20% and, a minimum power of 80%. This calculation defined 50 older women from each county.

The characterization of the sample was presented using measures of central tendency, variability, and frequency. The distribution of normality of the data was evaluated by the Anderson–Darling test, and the homogeneity of variances by the Levene test. In order to verify the difference in plasma levels of inflammatory mediators (IL-6 and sTNFR-1) and functional capacity (SPPB) among the older women living in the three counties, one-way analysis of variance was performed (ANOVA one-way) with Bonferroni post hoc correction for normal distribution data and the Kruskal–Wallis test with Mann–Whitney post hoc for data with nonnormal distribution. *p* values <0.05 were considered statistically significant. The statistical analysis was performed using SPSS (version 20.0 for Windows).

## 3. Results

### 3.1. Participant Characteristics

**Figure 1:**
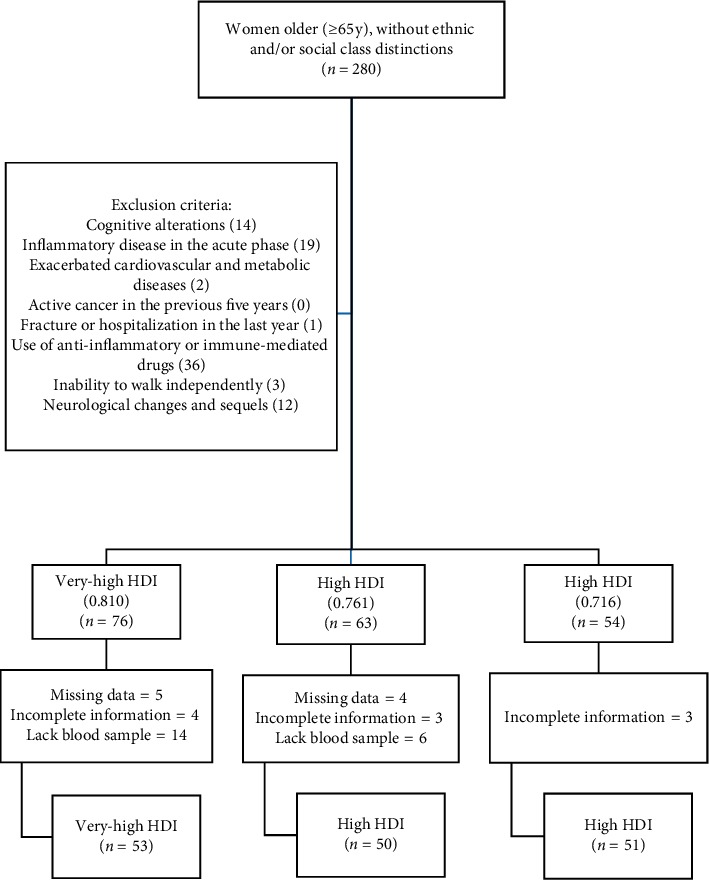
Flowchart demonstrating sample selection and inclusion and exclusion criteria.

A total of 154 community-based older women were enrolled in this study: 53 (73.17 ± 6.29 years) were from the county with the highest HDI (0.810) and the rest from other counties with high HDI (*n* = 50, 72.08 ± 5.50 years (HDI 0.761) and *n* = 51, 72.92 ± 6 years (HDI 0.716), respectively). (Figure 1).

The clinical and sociodemographic characteristics of the older women in the three counties are shown in Table 1. There was a significant difference among the older women in the three counties in educational level (*p* < 0.01), family income (*p* < 0.01), and number of diseases (*p* < 0.02), confirming the differences regarding the HDI. Life satisfaction was also different between the two groups (*p* < 0.01) showing lower satisfaction in the county with very high HDI. Other comparisons of clinical and sociodemographic variables resulted not being significant (*p* > 0.05) (Table 1).

### 3.2. Functional Capacity

There was a statistical difference in functional capacity (SPPB) showing that one of the counties with high HDI was in worse condition than the others (*p* < 0.001) (Table 2).

### 3.3. IL-6 and sTNFR-1

Differences in IL-6 plasma levels were not significant among the counties (*p* > 0.265). Regarding sTNFR-1 plasma levels, there was a significant difference between the counties (*p* < 0.001). Older women in the counties with high HDI had higher levels of sTNFR-1 compared with those who lived in the county with very high HDI (Table 2).

## 4. Discussion

This study aimed to compare the plasma levels of inflammatory mediators (IL-6 and sTNFR-1) and functional capacity among community-dwellings of older women living in counties with different HDI. The clinical and sociodemographic characteristics of the studied population is in line with other literature findings, showing a high percentage of widows, with low level of education and low income, presence of comorbidities, and use of long-term medications [1, 15]. There was a difference between the counties in relation to level of education, family income, and number of self-reported diseases confirming the presence of regional diversities in the aging process [5, 11], in accordance with HDI criteria. When comparing the characteristics of the population, it was expected that only variables related to the HDI domains would be different. However, there was a difference regarding life satisfaction information, showing that older women living in one of the lower HDI cities reported greater satisfaction with life. In the same city, there was the lowest functional capacity score and the highest indices of sTNFR-1.

Regarding functionality, the presence of multiple diseases and differences in the socioeconomic situation seem to play a central role in the health of older women, due to the influence on the demand for health services, less information about prevention, and poor health-related life habits. Thus, it can be thought that the low educational condition and the greater number of chronic autoreported diseases could be contributing to the worse functionality of these older women [3–5, 16]. However, this does not appear to have influenced life satisfaction, probably due to greater resilience and less knowledge of the opportunities of a more developed city. Thus, it is believed that the lack of information on preventive measures, as well as the lack of access to health centers and physical activities, as occurs in large cities, could contribute to this decrease in functional capacity. Torres et al. [16] showed an association between levels of income, functionality, and health conditions, the latter being evaluated through the presence of chronic diseases and self-perceived health [16]. Although verifying associations was not the objective of this study, the presence of differences among the counties pointed to possible influences of these variables on functional capacity and plasma levels of inflammatory mediators. On the other hand, it can be hypothesized that inadequate living habits could impact the health and functionality of the elderly. These associations could be explored in future studies.

In addition, there is evidence that adverse socioeconomic, behavioral, and environmental conditions can lead to changes in the immune system of the older person, with alteration in the production and expression of proinflammatory cytokines. These authors showed that high levels of inflammatory mediator indices were associated with low socioeconomic conditions but did not make comparisons between specific groups [17, 18]. In addition, other researchers have demonstrated the effect of social inequality on psychobiological processes, inferring that psychosocial factors would stimulate biological systems through the central nervous system, triggering autonomic, neuroendocrine, and immunological responses [19]. However, this assumption cannot be supported by this study, as these variables were not researched, which could be the subject of future studies.

In the present study, the IL-6 plasma levels of the older women evaluated were not different among the three counties. Felicio et al. [8] found that IL-6 plasma levels below the median of 0.87 pg/mL would probably not result in adverse effects on muscle function and physical performance. The authors also indicated that changes in inflammatory cytokines would occur at the cellular level even before adverse results appear, i.e., high levels of cytokines would only become apparent in the long term [8]. In addition, Barbieri et al. [20] observed adverse effects of IL-6 only at plasma concentrations above 1.73 pg/mL [20]. In the present study, IL-6 plasma concentrations of the older women in the counties with a high HDI reached a median of 1.78 pg/mL, which, supported by the literature, is considered a sufficient concentration to cause adverse events. Thus, it can be hypothesized that the higher concentrations of IL-6 of these older women could contribute to the greater number of diseases and worse functionality. In this sense, it is suggested to carry out new studies to investigate possible cutoff points in order to consider high plasma levels of IL-6 in specific populations. It should also be considered that one of the explanations for the absence of differences in IL-6 levels among the older women in the counties studied could be the large variability of this cytokine.

Moreover, comparing the levels of sTNFR-1, we observed a higher concentration of this cytokine in older women from the counties with high HDI compared to those with very high HDI. Current evidence indicates that chronically elevated levels of inflammatory cytokines, such as TNF-*α*, would cause muscle catabolism [21]. Inflammatory cytokines can inhibit the synthesis of muscle proteins, accelerate protein decomposition, and upregulate the expression of muscle growth inhibitory factor myostatin and muscle atrophy proteins, so as to accelerate protein catabolism. Insulin resistance is another mechanism of loss of muscle mass and strength. In this way, elevated IL-6 and TNF-*α* serum levels can lead to insulin resistance and the occurrence of loss of muscle mass and function [22]. In human studies, there was a positive correlation between protein degradation and TNF-*α* production and a negative correlation between heavy chain myosin protein rates and expression of TNF-*α*, IL-6, and C-reactive protein [23, 24]. In addition, it is known that the subliminal process of chronic inflammation occurs even without the presence of disease manifestations but contributes to its onset, such as sarcopenia, leading to loss of functionality [23, 24]. In this sense, from these results, it can be expected that the higher levels of sTNFR-1 could be related to the higher number of self-reported illnesses and the low functional condition presented in the women of the high HDI counties.

This assumption can be explained by a spiral effect. Thus, it is important to emphasize that women live longer with chronic-degenerative diseases. Similarly, considering the relationship between neuroendocrine and immunological dysregulation, which occurs in aging, it can be thought that these women were more susceptible to the influence of alterations of the pituitary adrenal system [20, 24, 25]. In addition, impairments in mitochondrial turnover have recently emerged as an additional mechanism linking inflammation to mitochondrial dysfunction [26]. However, further research is necessary to understand the complex interaction between mitochondrial dysfunction, inflammation, and functional capacity [25, 26]. Finally, several studies have shown only the relationship or difference of one or two inflammatory mediators, especially IL-6 and TNF-*α* [25], as in the present study. However, the inflammatory system is highly complex and involves several cellular components of the innate and adaptive immune response [25, 26], that should be better explored.

Some limitations should be considered. We only studied a female population, supported by the process of “feminization of old age” and the disparities in the aging process between men and women. We believe this was a sensible choice, despite the limitations in relation to the generalization of the results. From a clinical applicability perspective, the results mainly demonstrated the need for greater attention to functional modifications among the elderly, considering their socioeconomic and environmental context.

## 5. Conclusion

The results showed that older women from the county with very high HDI had lower sTNFR-1. Functional capacity was lower in one of the counties with lower HDI. Also, the results confirmed the differences between the counties in relation to levels of education, family income, and number of self-reported diseases, according to the HDI. Further studies are suggested to verify the association of these factors with differences in sTNFR-1 and functional capacity.

## Figures and Tables

**Table 1:** Clinical and sociodemographic characteristics of the older women divided by counties and *p* value.

Variable	HDI very high (0.810) (*n* = 53)	HDI high (0.761) (*n* = 50)	HDI high (0.716) (*n* = 51)	*p* value
Age, years, average (SD)	73.2 (6.3)	72.1 (5.5)	72.9 (6.2)	0.80
Education level, years, average (SD)	6.0 (4.3)	2.7 (3.0)	2.7 (1.0)	<0.01^*∗*^
Income, salary, average (SD)	1.6 (1.2)	2.0 (0.7)	1.3 (0.5)	<0.01^*∗*^
Diseases, number, average (SD)	2.8 (2.0)	4.0 (2.6)	2.7 (1.5)	<0.02^*∗*^
Medication, number, average (SD)	3.3 (2.4)	4.3 (3.5)	2.9 (1.9)	0.62
Intensity of pain, average (SD)	6.6 (2.8)	6.2 (2.3)	6.3 (2.4)	0.73
BMI, kg/m^2^, average (SD)	27.0 (4.3)	27.6 (5.9)	26.7 (4.9)	0.83

Marital status				0.43
Married (%)	26.4	44.0	41.2	
Single (%)	20.8	10.0	11.8	
Divorced (%)	13.2	8.0	7.8	
Widow (%)	39.6	38.0	39.2	

Race				0.16
White (%)	39.6	64.0	39.2	
Black (%)	11.3	8.0	15.7	
Mixed (%)	47.2	28.0	43.1	
Oriental (%)	1.9	—	2.0	

Self-perception of health				0.07
Bad (%)	9.4	2.0	5.9	
More or less (%)	35.8	54.0	60.8	
Good (%)	54.7	44.0	33.3	

Satisfaction with life				<0.01^*∗*^
Lithe (%)	11.3	—	—	
More or less (%)	24.5	22.0	35.3	
Much (%)	64.2	78.0	64.7	

Presence of pain				0.43
No (%)	35.8	36.0	25.5	
Yes (%)	64.2	64.0	74.5	

Smoking				0.73
No (%)	92.5	80.0	96.1	
Yes (%)	7.5	20.0	3.9	

Alcoholism				0.32
No (%)	93.0	74.0	96.0	
Yes (%)	7.0	26.0	4.0	

BMI = body mass index; SD = standard deviation; ^*∗*^statistical difference by the Kruskal–Wallis test.

**Table 2:** Comparison of functional capacity and plasma levels of IL-6 and sTNFR-1 among the participating women considering the different counties.

Variable	HDI very high (0.810) (*n* = 53)	HDI high (0.761) (*n* = 50)	HDI high (0.716) (*n* = 51)	*p* value
SPPB, score, median, interquartile range	10.0 (8.0–11.0)	6.0ᵵ (5.0–7.0)	10.0 (8.0–11.0)	<0.01^≈^
IL-6, pg/mL, median, interquartile range	1.4 (0.8–2.1)	1.8 (1.0–2.6)	1.5 (1.1–2.0)	0.27
sTNFR-1, ng/mL, median, interquartile range	150.6ᵵ (130.0–178.9)	189.1 (155.2–224.3)	183.0 (159.6–183.0)	<0.01^*∗*^

SPPB = short physical performance battery; IL-6 = interleukin-6; sTNFR-1 = soluble receptor of tumor necrosis factor 1; ^*∗*^statistical difference by the ANOVA one-way test; the difference observed by the Bonferroni test; statistical difference by the Kruskal–Wallis test; ᵵ difference observed by the Mann-Whitney *U* test; ^*∗*^statistical difference by the Kruskal–Wallis test; ᵵ difference observed by the Mann–Whitney *U* test.

## Data Availability

The data used to support the findings of this study are available from the corresponding author upon request.
